# Severe Traumatic Injury Induces Phenotypic and Functional Changes of Neutrophils and Monocytes

**DOI:** 10.3390/jcm10184139

**Published:** 2021-09-14

**Authors:** Andrea Janicova, Nils Becker, Baolin Xu, Marija Simic, Laurens Noack, Nils Wagner, Andreas J. Müller, Jessica Bertrand, Ingo Marzi, Borna Relja

**Affiliations:** 1 Experimental Radiology, Department of Radiology and Nuclear Medicine, Otto-von-Guericke-University Magdeburg, Leipziger Straße 44, 39120 Magdeburg, Germany; andrea.janicova@med.ovgu.de (A.J.); nibecker@ukaachen.de (N.B.); xubaolin325@outlook.com (B.X.); marija.simic@med.ovgu.de (M.S.); laurens.noack@st.ovgu.de (L.N.); 2 Department of Trauma, Hand and Reconstructive Surgery, Goethe University, Theodor-Stern-Kai 7, 60590 Frankfurt am Main, Germany; nils.wagner@kgu.de (N.W.); ingo.marzi@kgu.de (I.M.); 3 Department of Trauma Surgery, Hospital of the RWTH University, Pauwelsstraße 30, 52074 Aachen, Germany; 4 Institute of Molecular and Clinical Immunology, Health Campus Immunology Infectiology and Inflammation, Otto-von-Guericke-University Magdeburg, Leipziger Straße 44, 39120 Magdeburg, Germany; andreas.mueller@med.ovgu.de; 5 Intravital Microscopy in Infection and Immunity, Helmholtz Centre for Infection Research, Inhoffenstraße 7, 38124 Braunschweig, Germany; 6 Department of Orthopaedic Surgery, Otto-von-Guericke University Magdeburg, Leipziger Straße 44, 39120 Magdeburg, Germany; jessica.bertrand@med.ovgu.de

**Keywords:** traumatic injury, reactive oxygen species, phagocytosis, CD14, CD16, CD62L, fMLP, PMA

## Abstract

Background: Severe traumatic injury has been associated with high susceptibility for the development of secondary complications caused by dysbalanced immune response. As the first line of the cellular immune response, neutrophils and monocytes recruited to the site of tissue damage and/or infection, are divided into three different subsets according to their CD16/CD62L and CD16/CD14 expression, respectively. Their differential functions have not yet been clearly understood. Thus, we evaluated the phenotypic changes of neutrophil and monocyte subsets among their functionality regarding oxidative burst and the phagocytic capacity in severely traumatized patients. Methods: Peripheral blood was withdrawn from severely injured trauma patients (TP; *n* = 15, ISS ≥ 16) within the first 12 h post-trauma and from healthy volunteers (HV; *n* = 15) and stimulated with fMLP and PMA. CD16^dim^CD62L^bright^ (immature), CD16^bright^CD62L^bright^ (mature) and CD16^bright^CD62L^dim^ (CD62L^low^) neutrophil subsets and CD14^bright^CD16^−^ (classical), CD14^bright^CD16^+^ (intermediate) and CD14^dim^CD16^+^ (non-classical) monocyte subsets of HV and TP were either directly analyzed by flow cytometry or the examined subsets of HV were sorted first by fluorescence-activated cell sorting and subsequently analyzed. Subset-specific generation of reactive oxygen species (ROS) and of *E. coli* bioparticle phagocytosis were evaluated. Results: In TP, the counts of immature neutrophils were significantly increased vs. HV. The numbers of mature and CD62L^dim^ neutrophils remained unchanged but the production of ROS was significantly enhanced in TP vs. HV and the stimulation with fMLP significantly increased the generation of ROS in the mature and CD62L^dim^ neutrophils of HV. The counts of phagocyting neutrophils did not change but the mean phagocytic capacity showed an increasing trend in TP. In TP, the monocytes shifted toward the intermediate phenotype, whereas the classical and non-classical monocytes became less abundant. ROS generation was significantly increased in all monocyte subsets in TP vs. HV and PMA stimulation significantly increased those level in both, HV and TP. However, the PMA-induced mean ROS generation was significantly lower in intermediate monocytes of TP vs. HV. Sorting of monocyte and neutrophil subsets revealed a significant increase of ROS and decrease of phagocytic capacity vs. whole blood analysis. Conclusions: Neutrophils and monocytes display a phenotypic shift following severe injury. The increased functional abnormalities of certain subsets may contribute to the dysbalanced immune response and attenuate the antimicrobial function and thus, may represent a potential therapeutic target. Further studies on isolated subsets are necessary for evaluation of their physiological role after severe traumatic injury.

## 1. Introduction

Severe traumatic injury is with 5.8 million annual deaths one of the most common causes of death worldwide [1]. Although the survival rates have improved globally in the past decades due to advanced post-traumatic treatment, the development of immune-related secondary complications such as systemic inflammatory response syndrome, nosocomial infections, or sepsis, remains the major contributing factor in trauma-associated mortality [2,3,4]. Approximately 55% of polytrauma patients suffer from severe chest injury, leading to a direct lung tissue damage on the one hand and to activation of inflammatory response on the other hand, resulting in alveolocapillary membrane breakdown [5]. These patients are highly susceptible for disseminated intravascular coagulation, pneumonia, as well as acute lung injury, and its more severe form acute respiratory distress syndrome (ARDS) [5]. ARDS is associated with a dysregulated immune response, an overall mortality between 35% and 50%, and treatment of those patients is limited [6]. An excessive generation of ROS by the injured endothelium and epithelium as well as recruited leukocytes play a major role in ARDS progression and lung damage [7]. Moreover, a dysregulated immune response after trauma has been associated with post-traumatic phenotypical and functional aberrations of certain cells of the innate immune system such as neutrophils and monocytes [7,8,9,10].

Neutrophils and monocytes represent the first line of immune defense following an infection or a sterile injury [11,12]. Both are released from the bone marrow into the circulation and are recruited to sites of infection or tissue damage towards the pathogen-derived signaling molecules (pathogen-associated molecular patterns) or host-produced inflammatory signaling molecules (damage-associated molecular patterns), respectively [11,12]. The ability to recognize and eliminate bacteria and debris is critical for timely resolution of inflammation and recovery from the injury [13,14]. An impairment of phagocytic activity ex vivo has been observed in patients with community-acquired pneumonia [15], spinal cord injury [16] and traumatic brain injury [17,18] compared to healthy individuals. Moreover, neutrophil depletion impedes the clearance of the debris from necrotic sites in sterile hepatic injury, leading to impairment of regeneration and revascularization of the focal injury [10]. As some neutrophils in human peripheral neutrophil pool phagocyte their targets better than others [19], certain neutrophil populations may have differential functionality in this process.

Although the production of reactive oxygen species (ROS) is essential for the pathogen clearance, an exaggerated release of proteases and oxygen radicals may lead to collateral tissue damage and contribute to the development of secondary complications [20,21]. In the context of acute lung injury or its most severe form ARDS, excessive generation of ROS leads to the loss of junctional integrity of vascular microvessels, promoting the migration of polymorphonuclear leukocytes and the transition of fluids in the alveolar lumen, causing pulmonary edema [7].

Three different subsets of neutrophils were described according to their expression of the cluster of differentiation (CD)16 and CD62L expression [22]. CD16^dim^CD62L^bright^ subset shows the characteristics of immature neutrophils with banded nuclei, CD16^bright^CD62L^bright^ population represents the mature neutrophils with prototypically segmented nuclei, and CD16^bright^CD62L^dim^ neutrophils are characterized by hypersegmented nuclei. The latter have been shown to directly suppress lymphocyte proliferation by ROS release into the immunological synapse between neutrophil and T-cell, suggesting its immunosuppressive features [22]. Although all neutrophil subsets enter the circulation almost immediately after trauma, their precise post-traumatic function still remains elusive [8].

Similarly, three different subsets of monocyte can be distinguished according to their CD16 and CD14 surface expression [23,24]. Classical monocytes, characterized by CD16^−^CD14^bright^ expression contribute to bacterial clearing and immune sensing, whereas non-classical CD16^+^CD14^dim^ monocytes patrol along the blood vessel walls [25]. Intermediate CD16^+^CD14^bright^ monocytes share particularly the functions of classical and non-classical subsets but have more pro-inflammatory features and have been linked to the regulation of apoptosis and transendothelial migration [23,24]. Following human experimental endotoxemia, all monocyte subsets are lost within one to two hours after lipopolysaccharide administration and recover from classical, over intermediate to non-classical monocytes within 8–24 h [9]. This clearly confirms the generally accepted theory, that monocyte subsets differentiate from classical to intermediate and non-classical monocytes in a dynamic process. There is no evidence about the subset dynamics including specific kinetics and functions following traumatic injury in humans.

Considering that a well-orchestrated immune response is essential for the initiation and subsequent resolution of inflammation and an adverse phenotype and functional transition of neutrophils and monocytes may negatively affect the post-traumatic outcome, we evaluated the phenotypic shift of neutrophils and monocytes as well as their phagocytic capacity and ROS production in the currently known subsets of those cell types.

We hypothesized that major traumatic injury shifts the cell subset ratios toward the pro-inflammatory phenotypes early after trauma and that along with this, the circulatory cells will exert modified functions regarding phagocytosis and oxidative burst.

## 2. Materials and Methods

### 2.1. Ethics

The current study was performed at the University Hospital of the Goethe-University Frankfurt in accordance with the Declaration of Helsinki and following the Strengthening the Reporting of Observational studies in Epidemiology-guidelines [26].

### 2.2. Patient Cohort

Fifteen healthy volunteers and 15 severely injured patients between 18 and 50 years of age were enrolled. Trauma patients with an injury severity score (ISS) ≥ 16 were included and the samples were collected and analyzed within the first 12 h post-injury. Exclusion criteria included an acute infection, pre-existing chronic inflammatory diseases, immunological disorders, human immunodeficiency virus infection, infectious hepatitis, immunosuppressive medication, and pregnancy.

### 2.3. Staining of Neutrophil and Monocyte Subsets for Flow Cytometry

The blood of trauma patients and healthy volunteers was withdrawn into Li-Heparin blood collection tube (Sarstedt, Nümbrecht, Germany), stored on ice and processed within 30 min. A total of 100 µL of heparinized blood was transferred into FACS tube (Corning, New York, NY, USA). For neutrophils, heparinized blood was stimulated with 1 × 10^−3^ mM N-formyl-methionyl-leucyl-phenylalanine (fMLP) on ice for 15 min. Subsequently, samples were washed with 2 mL ice-cold FACS buffer (0.5% bovine serum albumin in 1× phosphate-buffered saline (PBS) without Mg^2+^ and Ca^2+^), gently vortexed, and centrifuged at 350× *g* and at 4 °C for 7 min. After the supernatant was discarded, 5 µL Alexa Fluor 647-conjugated anti-human CD16 antibody (clone 3G8; BD Biosciences, Franklin Lakes, NJ, USA) and 20 µL PE-conjugated anti-human CD62L antibody (clone SK11; BD Biosciences, USA) were added. For monocytes, heparinized blood was stimulated with 1 × 10^−4^ mM phorbol 12-myristate 13-acetate (PMA) on ice for 15 min. Subsequently, the samples were washed. 5 µL Alexa Fluor 647-conjugated anti-human CD16 antibody (clone 3G8; BD Biosciences, USA) and 5 µL PE-conjugated anti-human CD14 antibody (clone M5E2; BioLegend, San Diego, CA, USA) were added, all samples gently vortexed and incubated in the dark on ice for 30 min. Subsequently, the samples were washed. Then, the protocols for functional analyses regarding the phagocytic capacity (see Section 2.4) and ROS generation (see Section 2.5) followed as described below.

### 2.4. Analysis of Bacterial Intake by Neutrophils by Flow Cytometry

FITC-labeled *E. coli* bioparticles were reconstituted in 1× PBS without Mg^2+^ and Ca^2+^ according to the manufacturer’s instructions (*Escherichia coli* (K-12 strain) BioParticles E-2861; Thermo Fisher Scientific, Waltham, MA, USA), aliquoted and stored at −20 °C and in dark until use. In the experiment, 10 bioparticles per leukocyte were added to the sample and incubated at 37 °C and 5% CO_2_ for 60 min. Following “bacterial” loading, cells were washed with 2 mL ice-cold FACS buffer and centrifuged at 350× *g* and at 4 °C for 7 min. For red blood cells lysis, 2 mL cold lysis buffer were added, and the samples were incubated in the dark at 4 °C for 10 min. Washing step with 2 mL ice-cold FACS buffer was repeated. Cells were resuspended in 500 µL ice-cold FACS buffer and immediately evaluated by flow cytometry. Granulocytes were defined by gating the corresponding forward and side scatter scan. From each sample a minimum of 5.0 × 10^4^ cells were measured, which were subsequently analyzed. The percentage of CD16^dim^CD62L^bright^ (immature), CD16^bright^CD62L^bright^ (mature) and CD16^bright^CD62L^dim^ (hypersegmented) neutrophils as well the percentage and mean fluorescent units of phagocytosis-positive out of the respective subsets were assessed by flow cytometric analyses using a BD FACSCanto 2™ and FACS DIVA™ software (BD Biosciences, USA).

### 2.5. Analysis of Reactive Oxygen Species Production in Neutrophils and Monocytes by Flow Cytometry

Totals of 90 µL warm PBS and 2 µL 100 µM CM-H _2_DCFDA (Thermo Fisher Scientific, USA) were added to samples (see Section 2.3) and incubated at 37 °C and 5% CO_2_ for 30 min. 2 mL warm RPMI 1640 medium (Gibco, Carlsbad, CA, USA), supplemented with 10% heat-inactivated fetal calf serum (Gibco, Carlsbad, CA, USA), 100 IU/mL penicillin (Gibco, USA), 10 μg/mL streptomycin (Gibco, Carlsbad, CA, USA) and 20 mM HEPES buffer (Sigma Aldrich, St. Lois, MO, USA) were added and samples were centrifuged at 350× *g* and at room temperature for 7 min. Supernatant was discarded and subsequently, 1 mL medium with supplements was added and the samples were incubated at 37 °C and 5% CO_2_ for further 60 min. Following the recovery step, samples were centrifuged at 350× *g* and at room temperature for 7 min and the supernatant was discarded. For red blood cells lysis, 2 mL lysis buffer (0.155 M NH_4_Cl, 0.01 M KHCO_3_, 0.1 mM ethylenediaminetetraacetic acid in distilled water) were added and incubated in the dark at 4 °C for 10 min. Washing step with 2 mL ice-cold FACS buffer was repeated. Cells were resuspended in 500 µL ice-cold FACS buffer. Granulocyte and monocyte populations were defined by gating the corresponding forward and side scatter scan. Their ratio out of leukocyte population was assessed after excluding the cell debris. From each sample a minimum of 5.0 × 10^4^ cells were measured, which were subsequently analyzed. For neutrophils, the percentage of CD16^dim^CD62L^bright^ (immature), CD16^bright^CD62L^bright^ (mature) and CD16^bright^CD62L^dim^ (CD62L^dim^) neutrophils and for monocytes, the percentage of CD14brightCD16^−^ (classical), CD14^bright^CD16^+^ (intermediate) and CD14^dim^CD16^+^ (non-classical) as well the percentage and mean fluorescent units of ROS-positive out of the respective subsets were assessed by flow cytometric analyses using a BD FACS Canto 2™ and FACS DIVA™ software (BD Biosciences, USA).

### 2.6. Fluorescence-Activated Cell Sorting of Neutrophil and Monocyte Subsets

For each neutrophil and monocyte subsets, the blood of healthy volunteers was freshly withdrawn into Li-Heparin blood collection tubes (Sarstedt, Germany), stored on ice and processed within 30 min. For each neutrophil subset, 200 µL of heparinized blood was transferred into FACS tube (Corning, USA). For neutrophils, 10 µL Alexa Fluor 647-conjugated anti-human CD16 antibody (clone 3G8; BD Biosciences, USA) and 40 µL PE-conjugated anti-human CD62L antibody (clone SK11; BD Biosciences, USA) were added. The sample was gently mixed and incubated in the dark and on ice for 30 min. For washing, 2 mL ice-cold FACS buffer were added, and samples were subsequently centrifuged at 350× *g* and at 4 °C for 7 min. The supernatant was discarded. For red blood cells lysis, 2 mL of cold red blood cell lysis buffer were added and incubated in the dark at 4 °C for 10 min. Washing step with 2 mL ice-cold FACS buffer was repeated. Cells were resuspended in 1000 µL ice-cold FACS buffer and immediately sorted by using BD FACSCalibur (BD Biosciences, USA).

For monocyte subsets, peripheral blood mononuclear cells (PBMCs) were isolated first by a density-gradient centrifugation (Biocoll separation solution, 1.077 g/mL density; Biochrom, Berlin, Germany). Here, 10 mL of Biocoll separation solution (room temperature) were carefully overlaid with 10 mL of at room temperature tempered heparinized blood and centrifuged at 800× *g* and at room temperature for 25 min. PBMCs in the interphase were transferred into FACS tube and washed with 3 mL ice-cold FACS buffer. Remaining red blood cells were lysed by 500 µL cold red blood cell lysis buffer at 4 °C for 10 min. After further washing step, PBMCs were resuspended in 200 µL ice-cold FACS buffer. Next, 20 µL Alexa Fluor 647-conjugated anti-human CD16 antibody (clone 3G8; BD Biosciences, USA) and 20 µL PE-conjugated anti-human CD14 antibody (clone M5E2; BioLegend, USA) were added. The sample was gently mixed and incubated in the dark and on ice for 30 min. Subsequently, cells were washed with 2 mL ice-cold FACS buffer and resuspended in 1000 µL ice-cold FACS buffer and immediately sorted by using BD FACSCalibur (BD Biosciences, USA).

Granulocyte and monocyte populations were defined by gating the corresponding forward and side scatter scan and the doublets were excluded. Neutrophil subsets were sorted as CD16^bright^CD62L^bright^ (mature) and CD16^bright^CD62L^dim^ (CD62L^dim^) using BD FACSCalibur™ (BD Biosciences, USA). Monocyte subsets were sorted as CD14brightCD16^−^ (classical), CD14^bright^CD16^+^ (intermediate) and CD14^dim^CD16^+^ (non-classical). Cell number and cell viability of the sorted populations were determined by Türk’s solution exclusion assay (Merck, Darmstadt, Germany). Sorted populations were reanalyzed and typically >95% pure. The generation of ROS (see Section 2.4; excluding the red blood cells lysis step) and the phagocytic capacity (see Section 2.5; excluding the red blood cells lysis step) were assessed.

### 2.7. Statistics

GraphPad Prism 5.0 software (GraphPad Software Inc., San Diego, CA, USA) was used to perform the statistical analysis. Data are given as mean ± standard error of the mean (SEM). The differences between the healthy volunteers and trauma patients were analyzed by Mann–Whitney *U*-test. The Kruskal–Wallis test with a Dunn’s post hoc test was applied to compare the differences between the subsets. A *p*-value below 0.05 was considered statistically significant.

## 3. Results

### 3.1. Patient Cohort

Fifteen patients with severe trauma and 15 healthy volunteers were enrolled in this study. The mean age of the patients was 38.2 ± 4.99 years of age. Two thirds of patients were male. All patients were substantially injured with an ISS of 27.7 ± 2.27. The mean stay in the intensive care unit was 15.5 ± 4.15 days, and the total duration of the in-hospital stay was 29.0 ± 7.32 days. The mean time of artificial ventilation was 8.4 ± 2.97 days. No patients developed ARDS, sepsis, or died. One patient developed pneumonia two days after admission to emergency department. The mean count of leukocytes in blood was 8.81/nL ± 0.92. The mean ratios of granulocytes and monocytes out of leukocytes were 67.8% ± 3.79 and 6.8% ± 0.98, respectively in trauma patients and 43.4% ± 2.35 and 6.6% ± 0.36, respectively in healthy subjects. The data are summarized in Table 1.

### 3.2. Severe Trauma Modulates the Distribution of CD16^+^ Neutrophil Subsets

**Figure 1:**
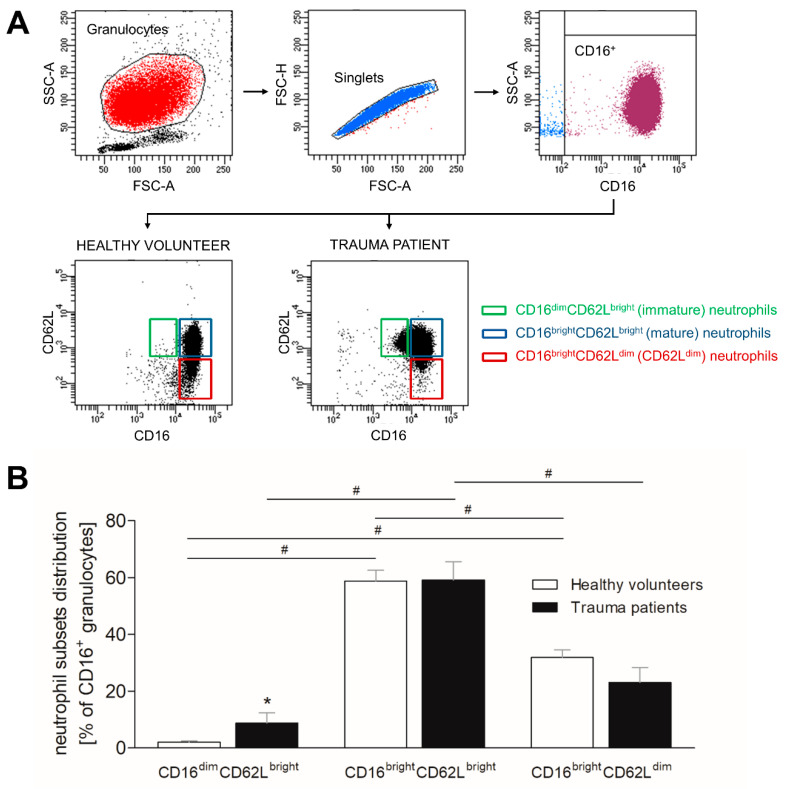
Impact of severe traumatic injury on neutrophil subset distribution. (**A**) Representative gating strategy for phenotyping human neutrophil subsets, including size discrimination, doublet exclusion, selection of CD16^+^ cells, and separation according to expression of CD16 and CD62L. (**B**) The percentage distribution of CD16^dim^CD62L^bright^ (immature), CD16^bright^CD62L^bright^ (mature) and CD16^bright^CD62L^dim^ (CD62L^dim^) neutrophils out of CD16^+^ granulocytes was determined in healthy subjects (white bars) and severely injured patients (black bars) within 12 h postinjury. Data are presented as mean ± standard error of the mean. *: *p* < 0.05 vs. healthy volunteers; #: *p* < 0.05 vs. respective subset.

As it is known that severe traumatic injury has modulating effects on the immune system, we investigated the distribution of three neutrophil subsets according their CD16 and CD62L expression. Circulatory neutrophils were stained ex vivo and evaluated by flow cytometry. The gating strategy as well as the representative figures of CD16^dim^CD62L^bright^ (immature), CD16^bright^CD62L^bright^ (mature) and CD16^bright^CD62L^dim^ (CD62L^dim^) neutrophil subsets in healthy volunteers and trauma patients are shown in Figure 1A. The main population of neutrophils in healthy volunteers and trauma patients is formed by mature neutrophils (Figure 1B). Immature and CD62L^dim^ neutrophil populations are significantly less present compared to mature neutrophils (Figure 1B, *p* < 0.05). Comparing healthy volunteers to severely injured patients, the numbers of immature neutrophils significantly increase following major injury (Figure 1B, *p* < 0.05), whereas the counts of mature neutrophils do not change, and a decreasing trend is shown in CD62L^dim^ neutrophil subset (Figure 1B).

### 3.3. Severe Traumatic Injury Causes a Phenotypic Shift of CD14^+^ Monocytes

**Figure 2:**
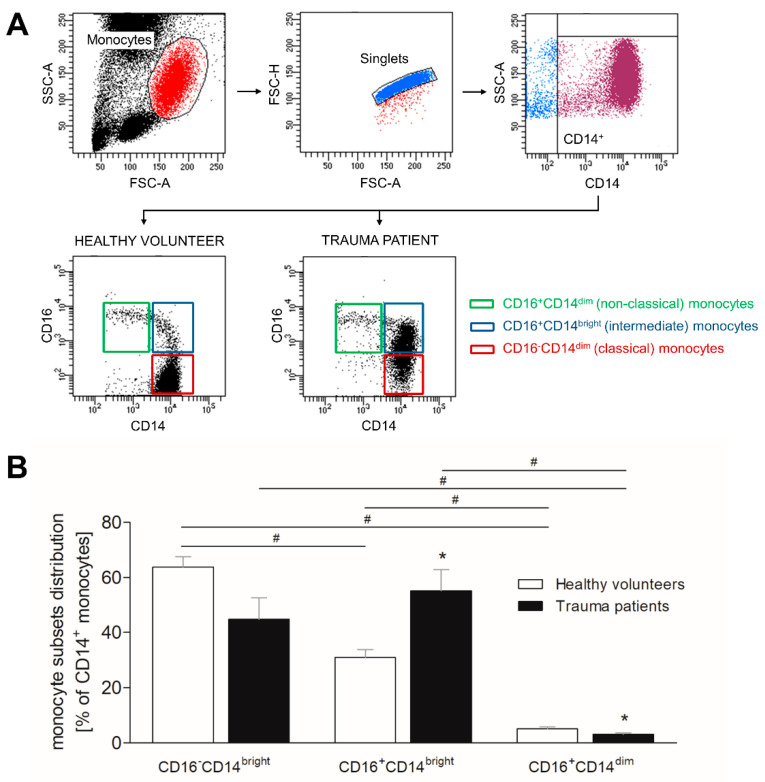
Impact of severe traumatic injury on monocyte subset distribution. (**A**) Representative gating strategy for phenotyping human monocyte subsets, including size discrimination, doublet exclusion, selection of CD14^+^ cells, and separation according to expression of CD16 and CD14. (**B**) The percentage distribution of CD14^bright^CD16^−^ (classical), CD14^bright^CD16^+^ (intermediate) and CD14^dim^CD16^+^ (non-classical) monocytes out of CD14^+^ monocytes was determined in healthy subjects (white bars) and severely injured patients (black bars) within 12 h postinjury. Data are presented as mean ± standard error of the mean. *: *p* < 0.05 vs. healthy volunteers; #: *p* < 0.05 vs. respective subset.

Similar to neutrophils, we evaluated the phenotypic redistribution of CD14^+^ monocytes following traumatic injury. The gating strategy as well as the representative figures of CD16^−^CD14^bright^ (classical), CD16^+^CD14^bright^ (intermediate) and CD16^+^CD14^dim^ (non-classical) monocyte subsets in healthy volunteers and trauma patients are shown in Figure 2A. In healthy volunteers, classical monocytes present the most abundant monocyte population, whereas intermediate and non-classical monocytes are significantly less present compared to this subset (Figure 2B, *p* < 0.05). The population of non-classical monocytes is also significantly smaller than the intermediate monocyte population (Figure 2B, *p* < 0.05). In trauma patients, intermediate subset presents the most abundant monocyte population and the classical monocyte counts have a decreasing trend compared to this subset (Figure 2B). Non-classical monocytes of trauma patients are significantly less present than classical and intermediate monocytes (Figure 2B, *p* < 0.05). Comparing healthy volunteers to trauma patients, classical monocyte population decreases in trauma patients, whereas intermediate monocyte population becomes significantly more abundant (Figure 2B, *p* < 0.05). The numbers of the non-classical monocytes significantly decrease in trauma patients (Figure 2B, *p* < 0.05).

### 3.4. Severe Trauma Does Not Affect Phagocytic Capacity of Neutrophils at Early Time Point

**Figure 3:**
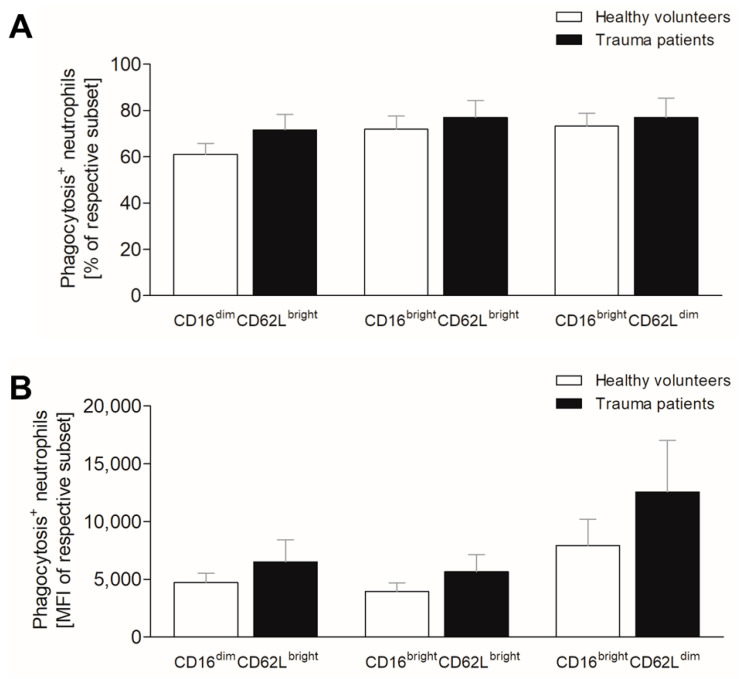
Impact of severe traumatic injury on phagocytic capacity of neutrophil subsets. The (**A**) percentage and (**B**) mean intensity of phagocytosis positive CD16^dim^CD62L^bright^ (immature), CD16^bright^CD62L^bright^ (mature) and CD16^bright^CD62L^low^ (CD62L^dim^) neutrophils were determined in healthy subjects (white bars) and severely injured patients (black bars) within 12 h postinjury. Data are presented as mean ± standard error of the mean.

Severely injured patients often develop secondary infectious complications, which may be caused by reduced phagocyting capacity [16]. Therefore, we evaluated the bacterial intake of neutrophils following traumatic injury. All three neutrophil populations obtained from healthy volunteers incorporate comparable numbers of FITC-labeled *E. coli* bioparticles, with slight increase in mature and CD62L^dim^ neutrophils, however, without significance (Figure 3A). Within the first 12 h after injury, the counts of phagocyting cells do not significantly change compared to respective subsets in healthy volunteers (Figure 3A).

The mean bioparticle intake per cell is comparable in immature and mature neutrophils of healthy volunteers, whereas the CD62L^dim^ neutrophils display higher phagocyting capacity compared to these two subsets (Figure 3B). Following traumatic injury, all three subsets exert increased mean phagocyting capacity without statistical significance compared to healthy volunteers (Figure 3B).

### 3.5. Severe Trauma Elevates the Production of Reactive Oxygen Species in Mature and CD62L^dim^ Neutrophils

**Figure 4:**
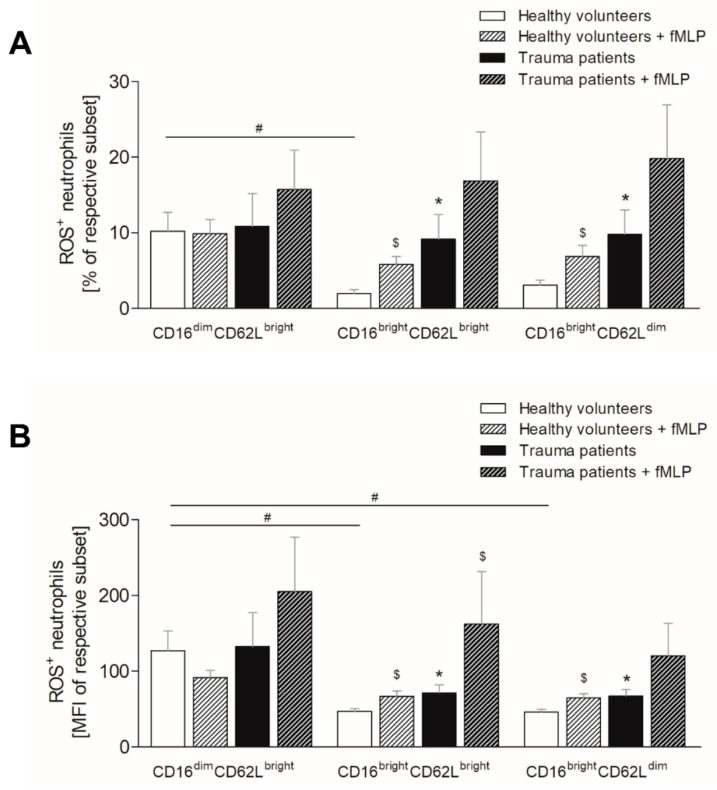
Impact of severe traumatic injury on generation of reactive oxygen species (ROS) by neutrophil subsets. The (**A**) percentage and (**B**) mean intensity of ROS positive CD16^dim^CD62L^bright^ (immature), CD16^bright^CD62L^bright^ (mature) and CD16^bright^CD62L^low^ (CD62L^dim^) neutrophils were determined in healthy subjects (white bars) and severely injured patients (black bars) within 12 h postinjury. Whole blood was stimulated with PMA ex vivo (diagonally striped bars). Data are presented as mean ± standard error of the mean. *: *p* < 0.05 vs. healthy volunteers; #: *p* < 0.05 vs. respective subset; $: *p* < 0.05 vs. unstimulated corresponding subset.

As it is known that enhanced ROS production contributes to endothelial dysfunction and tissue injury, we evaluated ROS levels in neutrophils. In healthy volunteers, significantly more immature neutrophils form ROS compared to mature and CD62L^dim^ neutrophils (Figure 4A, *p* < 0.05), whereas in trauma patients the counts of ROS positive neutrophils are equal in all three subsets (Figure 4A). Following traumatic injury, the counts of ROS positive immature neutrophils are comparable with these in healthy volunteers (Figure 4A), whereas the mature and CD62L^dim^ neutrophil subsets display significant increase of ROS positive neutrophils (Figure 4A, *p* < 0.05). Ex vivo stimulation of whole blood with fMLP does not affect the ratio of ROS positive neutrophils in the immature subset of healthy volunteers but significantly increases in the mature and CD62L^dim^ subset (Figure 4A, *p* < 0.05). In trauma patients, the fLMP-induced generation of ROS tends to increase compared to unstimulated samples, however, without significance (Figure 4A).

Similarly, the mean ROS production intensity per cell is the highest in immature neutrophil subset in both, healthy volunteers and trauma patients (Figure 4B). Those levels are significantly lower in mature and CD62L^dim^ neutrophils of trauma patients compared to immature neutrophils (Figure 4B, *p* < 0.05) and the levels in healthy volunteers are clearly lower as well, however, without statistical significance (Figure 4B). Comparing the ROS levels in trauma patients to healthy volunteers within the individual subsets, the mean ROS production of immature neutrophils does not change following traumatic injury (Figure 4B), whereas the levels in mature and CD62L^dim^ neutrophil population significantly increase (Figure 4B, *p* < 0.05). Similar to ROS positive neutrophils, the mean ROS generation intensity per cell does not change in the immature neutrophils of healthy volunteers following ex vivo fMLP stimulation and is significantly higher in mature and CD62L subsets (Figure 4B, *p* < 0.05). fMLP stimulation of trauma patient’s blood increases the mean generation of ROS compared to that of healthy subjects in the mature neutrophils, whereas the immature and CD62L^dim^ subsets show only increasing tendency (Figure 4B, *p* < 0.05).

### 3.6. Severe Traumatic Injury Increases the Production of Reactive Oxygen Species in Monocytes

**Figure 5:**
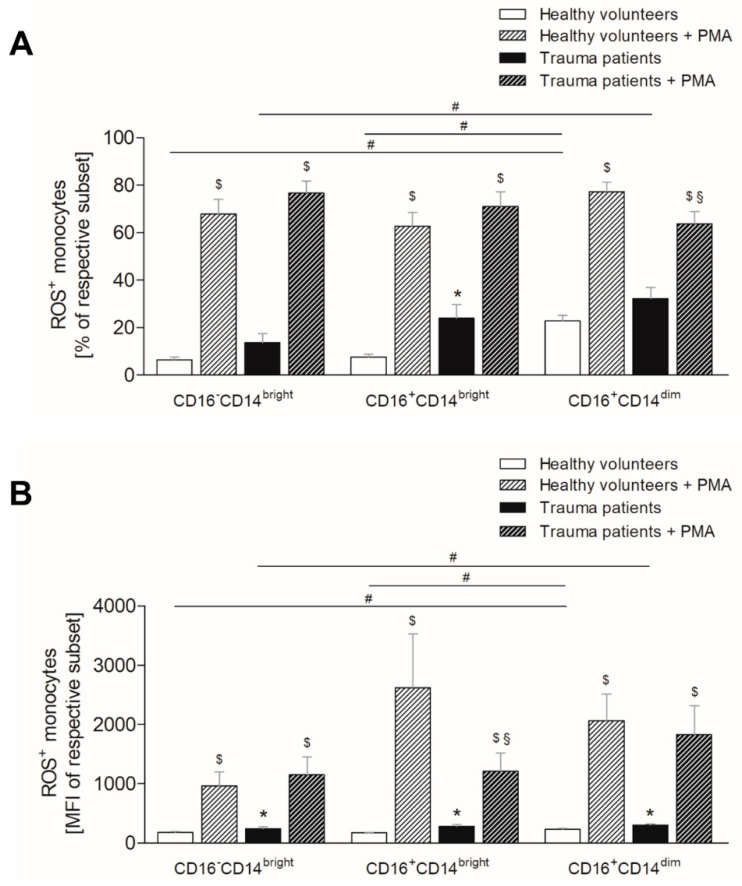
Impact of severe traumatic injury on generation of reactive oxygen species (ROS) by monocyte subsets. The (**A**) percentage and (**B**) mean intensity of ROS positive CD14brightCD16^−^ (classical), CD14^bright^CD16^+^ (intermediate) and CD14^dim^CD16^+^ (non-classical) monocytes were determined in healthy subjects (white bars) and severely injured patients (black bars) within 12 h postinjury. Whole blood was stimulated with PMA ex vivo (diagonally striped bars). Data are presented as mean ± standard error of the mean. *: *p* < 0.05 vs. healthy volunteers; #: *p* < 0.05 vs. respective subset; $: *p* < 0.05 vs. unstimulated corresponding subset; §: *p* < 0.05 vs. healthy volunteers + PMA.

Similar to neutrophils, monocytes display subset specific differences in ROS production following traumatic injury. In healthy volunteers, the same ratio of classical and intermediate monocytes generates ROS, whereas non-classical monocyte population displays significantly higher number of ROS positive cells (Figure 5A, *p* < 0.05). The monocytes obtained from trauma patients have increasing ratios of ROS positive cell from classical over intermediate to non-classical monocytes, whereby the difference between classical and non-classical subset is significant (Figure 5A, *p* < 0.05). When comparing trauma patients with healthy volunteers, there is an increasing ratio of ROS positive monocytes in all subsets, with significance in the intermediate subset (Figure 5A, *p* < 0.05). Ex vivo stimulation of whole blood of both healthy and injured subjects with PMA significantly increases the ratio of ROS positive monocytes within all three subsets compared to the unstimulated samples (Figure 5A, *p* < 0.05). However, only the non-classical monocytes of injured patients generate significantly less ROS following PMA stimulation compared to the equivalent subsets in healthy subjects (Figure 5A, *p* < 0.05).

The mean ROS production per cell is the highest in the non-classical subset, whereas classical and intermediate monocyte population is significantly less positive for ROS compared to non-classical monocytes (Figure 5B, *p* < 0.05). In trauma patients, classical monocytes produce significantly less ROS than non-classical monocytes (Figure 5B, *p* < 0.05), whereas the mean ROS production intensity of intermediate subset is comparable with that of non-classical monocyte population (Figure 5B). Comparing the respective subsets, all monocyte populations display significantly increased mean ROS production intensity following traumatic injury compared to healthy volunteers (Figure 5B, *p* < 0.05). Ex vivo stimulation with PMA leads similarly to the ratios of ROS positive monocytes, and also to a significant increase of mean ROS generation intensity in both healthy and injured subjects compared to untreated samples (Figure 5B, *p* < 0.05). This increase is significantly lower in the intermediate subset of severely injured patients compared to healthy subjects, whereas the mean intensity does not change in the classical and non-classical monocytes (Figure 5B, *p* < 0.05).

### 3.7. Fluorescence-Activated Cell Sorting Reveals an Exhaustion of Neutrophils and Monocytes and Aligns the Functional Differences between the Subsets

The individual neutrophil subsets of healthy volunteers were sorted by fluorescence-activated cell sorting (FACS) and subsequently analyzed for ROS production and phagocytic capacity. Nearly 100% of mature and CD62L^dim^ neutrophils were positive for ROS (Figure S1A) and the mean ROS production did not differ between the subsets as well (Figure S1B). Compared to whole blood analyses (see Figure 3), the production of ROS approximately increased 30-fold. The counts of phagocytosis positive neutrophils (Figure S1C) and the mean phagocytic capacity (Figure S1D) did not vary between the mature and CD62L^dim^ subsets. The bacterial intake is significantly impeded compared to whole blood analyses (see Figure 4). The immature neutrophil subset was not analyzed due to its low numbers and nearly absence in HV.

Similarly, nearly 100% of classical, intermediate, and non-classical monocytes were positive for ROS following FACS (Figure S2A). The mean production of ROS did not differ between the subsets (Figure S2B). Compared to whole blood analyses (see Figure 5), all monocyte subsets produce significantly higher amounts of ROS.

## 4. Discussion

Although the clinical treatment algorithms of severely injured trauma patients have improved over the last decades, leading to higher post-traumatic survival rates, those patients are still highly vulnerable to secondary infections during their clinical stay. The development of infectious complications has been associated with a dysregulated immune response, wherefore a mapping and characterization of immunological changes and intercellular interactions may predict post-traumatic vulnerability to secondary complications and potentially represent promising therapeutic targets in future [2,3,4]. Neutrophils and monocytes have been shown to initiate an immediate innate immune response with the aim to clear tissue damage and provide protection from invading pathogens [7,8,9,10]. Therefore, we investigated the phenotypical changes of different neutrophil and monocyte subsets in severely injured trauma patients as well as their functionality regarding the ROS generation and phagocytic capacity.

In this study, severely injured patients’ leukocyte counts remain in the normal range, whereas the ratio of granulocytes significantly increases compared to healthy subjects. Emergency granulopoiesis, which has been extensively described following traumatic injury and microbial challenge [27,28], is a part of the first line of cellular defense that crucially modulate subsequent repair processes after tissue damage [29]. However, on the other hand, an exaggerated release of neutrophils leads to bone marrow exhaustion and in turn, can impair the innate immune response to secondary hit such as surgery or infection [21].

Subset-specifically, we observed that in both, healthy volunteers and severely injured patients mature neutrophils (CD16^brigh^CD62L^bright^) represent the most abundant neutrophil subset. Although it has been reported that only a homogenous population of mature neutrophils can be found in healthy individuals, we detected additionally the CD62L^dim^ population (CD16^brigh^CD62L^dim^). Comparing healthy volunteers with severely injured patients, the counts of CD16^brigh^CD62L^dim^ neutrophils did not differ between those. A significantly increased frequency and absolute cell numbers of CD16^brigh^CD62L^dim^ neutrophils in trauma patients is extensively described in the literature and thus, contradictory to our results [30]. CD16^brigh^CD62L^dim^ neutrophils occur within the first hour after the injury [30] and locally release hydrogen peroxide into the immunological synapse between the neutrophils and T cells in Mac-1-dependend manner, leading to the suppression of T cell activation [22]. This suggests that a neutrophil subset with dimmer CD62L expression exhibits immunosuppressive features. Although CD62L is rapidly shed from the cell surface upon neutrophil activation [31], also changes in osmotic pressure, pH value, or hemodynamic shear stress can cause CD62L shedding [32,33,34]. Thus, regarding our results, CD16^brigh^CD62L^dim^ population in healthy volunteers may not constitute of CD62L^dim^ neutrophils but rather we might potentially detect the mature neutrophils that underwent CD62L shedding ex vivo caused by mechanical stress caused by the isolation and analysis methods. This, however, should be the case for both healthy donors and trauma patients.

Further, we observed a massive presence of immature (CD16^dim^CD62L^bright^) neutrophils in severely injured patients, whereas healthy individuals lack this population. It is assumed that immature neutrophils have impaired functional ability [21]. Moreover, Spijkerman et al. have shown that the ratio of immature neutrophils positively correlates with the severity of the injury and the development of infectious complication [28]. This indicates that immature neutrophils have an inadequate immune response towards traumatic stimuli depending on the injury severity and wherefore, the ratio of immature neutrophils may become a potent tool in the prediction of infectious complications following traumatic injury.

For a timely resolution of inflammation and recovery from injury, phagocytes such as neutrophils and monocytes recognize and eliminate microbes and cell debris [13,14]. Thus, if the phagocytes fail to engulf and clear their targets, the tissue inflammation is prolonged, causing tissue damage with subsequent infectious complications [10]. In the present study, we did not observe any significant differences between the severely injured patients and healthy individuals and the individual neutrophil subsets regarding the phagocytosis in the first twelve hours after trauma. Actually, the references about aberrated phagocytic capacity of neutrophils following traumatic injury are not consistent [17,35,36]. However, it must be considered that the most studies evaluate the intake of fluorochrome-conjugated bacteria or bacterial bioparticles by neutrophils and not directly their killing potential. That an accurate engulfment of bacteria does not necessarily mean that the pathogens are also adequate killed is underlined by the study by Leliefeld et al., analyzing the incorporation of bacteria and the capacity of killing the bacteria by neutrophils in human experimental endotoxemia model [37]. Interestingly, immature neutrophils exhibit a superior engulfment of bacteria and killing capacity. However, even though mature and CD62L^dim^ subsets incorporate bacteria at comparable level to immature neutrophils, they are incapable to kill the bacteria, that has been associated with higher intraphagosomal pH, subsequent intracellular bacterial growth and escape of the pathogens from the neutrophils [37]. This indicates that the above-described positive correlation between the elevated ratio of immature neutrophils and the development of inflammatory complications does not depend on defective phagocytosis. Moreover, as severe traumatic injury leads to neutrophilia with subsequent bone marrow exhaustion and the CD62L^dim^ subset appears within the first hour after the injury [21,27,28,30], this may together with the incapability of CD62L^dim^ neutrophils to adequately kill the bacteria [37] contribute to the development of infectious complications in later time course, because the immune system might not adequate respond to the bacterial escape from the CD62L^dim^ neutrophils.

The generation of ROS is an elemental mechanism to clear the pathogens within the phagosome, but it also acts as a chemoattractant for immune cells to clear and repair the tissue. However, an exaggerated release of free radicals can have detrimental consequences to the host such a loss of junctional integrity of vascular microvessels that contributes to the development of pulmonary edema and has been associated with the development of ARDS, systemic inflammatory response syndrome and multiorgan failure (MOF) [7,20,21,38,39,40]. Although the extent of the generation of ROS seems to be injury severity-dependent [17] and the contribution of neutrophil-induced exaggerated oxidative burst in the development of secondary complications after trauma is generally accepted, there is no evidence about neutrophil subset-specific generation of ROS.

We have shown that the oxidative burst significantly increases in mature and CD62L^dim^ neutrophils of severely injured patients, but its level remains stable in immature neutrophils compared to healthy individuals. However, immature neutrophils have the highest ratio of ROS compared to the other subsets. This supports the suggestion that immature neutrophils may be the key players in development of secondary post-traumatic complications. In line, the mean generation of ROS in the immature neutrophils noticeable increases in severely injured patients following ex vivo stimulation with N-formyl-methionyl-leucyl-phenylalanine (fMLP) compared to stimulated blood samples from healthy subjects. It has been shown that immature neutrophils are apoptosis resistant [41], whereas an exaggerated oxidative burst to secondary hit post-trauma has been associated with uncontrolled inflammatory response, resulting in endothelial permeability and tissue damage [42,43,44]. This along with our data indicates that the ROS-induced collateral tissue damage and subsequent infectious complications following traumatic injury could be primarily caused by immature neutrophil subset. Taken together, neutrophils undergo phenotypical and functional changes in severely injured patients dependently on the injury severity and contribute to the development of secondary complications. Therefore, a consequent monitoring of those during the whole period of hospital stay along with the incidence of infectious complications could provide data about the prediction of post-traumatic vulnerability to secondary infections.

Similar to neutrophils, monocytes have a multi-faced role in maintaining the tissue homeostasis and responding to inflammatory stimuli in order to clear the pathogens and cellular debris with subsequent restoration of tissue integrity [45]. However, the initial monocyte activation after trauma is rapidly followed by substantial paralysis of monocyte function, reflected by the decreased surface presentation of human leukocyte antigen (HLA)-DR [46,47]. Delayed recovery of HLA-DR expression and decreased release of pro-inflammatory mediators such as interleukin (IL)-1β, IL-6, IL-8 and tumor necrosis factor-α (TNF-α) have been associated with the development of secondary infections and MOF [46,47]. However, these studies focus mainly on aberrated monocyte functions from 24 h after trauma and there are no data about potential phenotypical changes of human monocytes following traumatic injury at early time point. Therefore, we analyzed the redistribution of monocyte subsets within the first twelve hours after severe trauma.

It is generally accepted that classical CD16^−^CD14^bright^ monocytes, which is the main monocyte population in healthy individuals, are precursors for pro-inflammatory intermediate CD16^+^CD14^bright^ monocytes that in turn differentiate into patrolling non-classical CD16^+^CD14^low^ monocytes [48]. In trauma patients, the numbers of circulating intermediate monocytes significantly increase, whereas we have observed a decrease of classical and non-classical subsets, suggesting a phenotype switch toward the pro-inflammatory phenotype. Classical monocytes have anti-microbial features with superior phagocytic capacity and after entering the tissues, they differentiate into monocyte-derived macrophages or dendritic cells [24]. Thus, the initial decrease of circulating classical monocytes after severe trauma might be caused on the one hand by a differentiation into intermediate monocytes and on the other hand by their transmigration to injury site in order to shape and resolve the inflammation. Regarding the intermediate monocytes, such an exaggerated elevation has been shown in severely injured patients 48 h post-trauma [49] and under inflammatory conditions such as sepsis or bacterial and viral infections [9], paralleled by sequestration of high amounts of TNF-α, IL-1β, and IL-6 [50]. Such an exaggerated release of pro-inflammatory cytokines has been associated with a so-called cytokine storm, that in turn can lead to blood pressure collapse, coagulopathy, up to MOF and death [51]. Therefore, the extent of the intermediate subset may prospectively provide an information about secondary post-traumatic complications.

Although an excessive oxidative burst has been already shown in monocytes of severely injured patients [52,53], there is no evidence whether the monocyte subsets generate ROS in a different extent and thus, may differently contribute to the pathogen and tissue clearance. In the present study, all monocyte subsets have significantly increased mean capacity to produce ROS compared to healthy individuals, which is comparable between the subsets. The ratio of ROS positive monocytes was elevated also in all subsets; however, a significant increase has been observed only in the pro-inflammatory intermediate monocytes. This along with the significant increase of the intermediate monocyte numbers support the assumption that the very early post-traumatic phase is characterized by a pro-inflammatory immune response of monocytes. Interestingly, ex vivo stimulation of whole blood with phorbol 12-myristate 13-acetate (PMA) leads to significant increase of mean generation of ROS compared to unstimulated samples, but this increase is significantly lower in the intermediate subset of severely injured patients compared to healthy volunteers. Once a pathogen is phagocytized, ROS contribute to the elimination of ingested pathogen and in the case of their not sufficient intracellular level, pathogen can escape and survive [54]. Thus, this inadequate intracellular oxidative burst of intermediate monocyte subset upon secondary stimuli may contribute to the susceptibility to infectious complications.

Interestingly, intermediate monocytes display the highest expression of HLA-DR in human experimental endotoxemia model compared to another two subsets over the whole observation period of 24 h [9]. Considering the post-traumatic monocyte deactivation within the first 48 h [9], it would be reasonable to follow the redistribution of monocyte subsets of severely injured patients and the subset-specific HLA-DR expression and the generation of ROS over the entire intensive care unit stay. We assume that the extent and the timing of the initial pro-inflammatory phase followed by the immunosuppressive phase in combination with the monocyte subsets distribution might provide valuable insight into post-traumatic monocyte kinetics and prospectively also a potent tool for counteracting the secondary infections.

For the evaluation of the physiological role of the neutrophil and monocyte subsets, studies on isolated subsets are necessary. Thus, we isolated the subsets by FACS. Although we obtained viable and clearly defined populations verified by flow cytometry, the above discussed functional differences between the subsets were no longer visible. Whereas nearly 100% of the cells were positive for oxidative burst and the mean levels elevated extraordinary, the bacterial incorporation was significantly impaired compared to whole blood analyses. We used BD FACSCalibur flow cytometer (BD Biosciences, USA), which is the first multicolor benchtop flow cytometry system capable of analyzing and sorting cells of interest for further study. The cell sorting rate and consequently the velocity, in which we obtained the requested cell counts, were extremely low. As both neutrophils and monocytes respond overly sensitive to their environment, so prolonged isolation led to cell exhaustion and impaired functionality. Thus, we have not been successful in isolating the individual subsets and subsequent analyzing their physiological roles and further studies under optimum conditions are necessary.

## 5. Limitations

This study provides a solid fundament for understanding the early post-traumatic phenotypic shift of neutrophils and monocytes and their antimicrobial functions, however, it also has several limitations. First, we only included fifteen polytrauma patients. Although the results shown are conclusive, increasing the number of study participants would enable a more precise group allocation according to the trauma pattern, and also correlation analyses of the evaluated neutrophil and monocyte subsets with clinical parameters such as ISS, bacterial complications, or ARDS. Second, we evaluated the phenotypic and functional changes only at the early time point. As severely injured patients develop secondary inflammatory complications in later time course, it would be reasonable to follow-up on the changes during the entire stay at intensive care unit. Third, as we did not use counting beads during the flow cytometry measurements, we obtained only relative fractions of neutrophil and monocyte subsets. Additionally, the percentage of neutrophils and monocytes out of leukocytes was evaluated by flow cytometry and, thus, this is not so precise as the blood analysis by hospital laboratory would be. Therefore, for achieving the absolute numbers, counting beads must be included in the upcoming studies as well as the blood work performed by hospital laboratory for evaluation of the ratios between the different leukocyte subpopulations. Lastly, FITC-labeled *E. coli* BioParticles were used for the phagocytic assay. This is a proper assay for the evaluation of bacterial intake; however, it is not possible to make a statement regarding the bacterial killing. A combination of the assessment of the bacterial intake and killing by neutrophils and monocytes would provide an immerse improvement of understanding the post-traumatic antimicrobial kinetics of leukocytes.

## 6. Conclusions

Severe traumatic injury induces an immediate phenotype shift of neutrophils as well as monocytes accompanied by their alterations in ROS generation compared to healthy subjects. In the circulation of trauma patients, the ratio of immature neutrophils is immensely elevated, whereas numbers of mature and CD62L^dim^ neutrophils do not change. All three subsets display an increasing tendency in phagocytic capacity and the mature and CD62L^dim^ neutrophils produce significantly more free radicals than those in healthy individuals. Thereby, an ex vivo stimulation with fMLP increases mean generation of ROS by trauma patients’ immature neutrophils compared to healthy subjects. Similarly, monocytes shift toward the pro-inflammatory intermediate phenotype and the classical and non-classical subsets becomes less abundant in trauma patients. All monocyte subsets generate high levels of ROS after severe traumatic injury. However, the intermediate and non-classical monocytes of severely injured patients generate significantly less ROS following ex vivo stimulation with PMA compared to healthy subjects. The here presented post-traumatic dynamic changes of those cells of innate immune system provide a solid fundament for functional studies of the individual subsets. The data following the ex vivo stimulation suggest that neutrophils may more contribute to the endothelial permeability and tissue damage, whereas monocytes seem to more contribute to higher susceptibility to secondary infections by their hyporesponsiveness to secondary hit. Future directions will include a larger cohort of severely injured patient and the analysis of trauma-induced phenotypical and functional changes of neutrophils and monocytes over an observation period of two weeks. We assume that the appearance and the antimicrobial functions of immature neutrophils and intermediate monocytes may be decisive for the development of secondary infectious complications in severely injured patients. The gained findings may improve the therapeutic approach or even contribute to a prevention of developing life-threatening infections.

## Figures and Tables

**Table 1:** Patient cohort.

	Parameter	SEM
Age	38.2 years old	4.99
Gender	10 men/5 women	-
ISS	27.7	2.27
Hospital stay	29.0 days	7.32
ICU stay	15.5 days	4.15
Ventilation	8.4 days	2.97
Death	0	-
Leukocytes	8.81/nL *	0.92
Neutrophils	67.8% of leukocytes	3.79
Monocytes	6.8% of leukocytes	0.98
Pneumonia	1 patient	-
ARDS	0	-
Sepsis	0	-

* Normal range: 3.92–9.81/nL. ARDS, acute respiratory distress syndrome; ICU, intensive care unit; ISS, injury severity score; SEM, standard error of mean.

## Data Availability

All data generated or analyzed during this study are included in this published article.

## Supplementary Materials

The following are available online at https://www.mdpi.com/article/10.3390/jcm10184139/s1, Figure S1: Generation of reactive oxygen species and phagocytic capacity in isolated neutrophil subsets obtained from healthy volunteers, Figure S2: Generation of reactive oxygen species in isolated monocyte subsets obtained from healthy volunteers.

## Abbreviations

ARDS	acute respiratory distress syndrome
CD	cluster of differentiation
FACS	fluorescence-activated cell sorting
fMLP	N-formyl-methionyl-leucyl-phenylalanine
IL	interleukin
ISS	injury severity score
MOF	multi organ failure
PBS	phosphate-buffered saline
PBMCs	peripheral blood mononuclear cells
PMA	phorbol 12-myristate 13-acetate
ROS	reactive oxygen species
