# Supplementary material for: Severe Traumatic Injury Induces Phenotypic and Functional Changes of Neutrophils and Monocytes
Source: J Clin Med. 2021 Sep 14;10(18):4139. doi: 10.3390/jcm10184139 (PMC8467869; doi:10.3390/jcm10184139)
Supplement: Supplementary file 1 [file jcm-10-04139-s001.zip › jcm-1382473-supplementary.pdf]

Journal of
Clinical Medicine

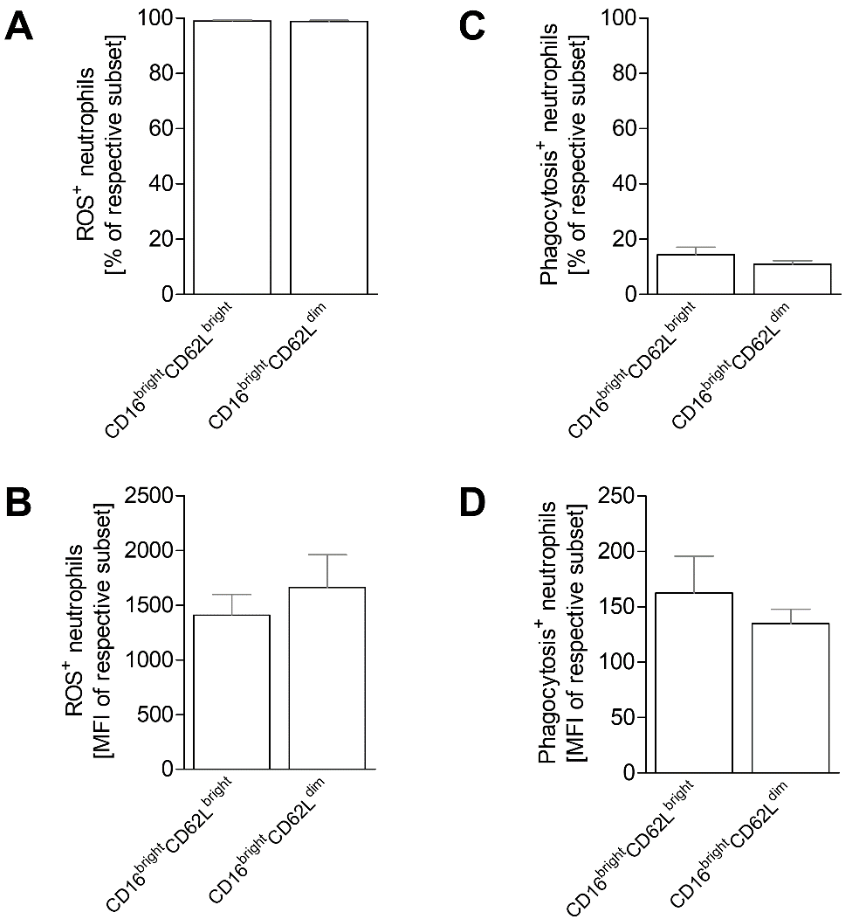

Figure S1. Generation of reactive oxygen species and phagocytic capacity in isolated neutrophil subsets obtained from severely injured patients. The $CD16^{dim}CD62L^{bright}$ (immature), $CD16^{bright}CD62L^{bright}$ (mature) and $CD16^{bright}CD62L^{dim}$ ($CD62L^{dim}$) neutrophils were isolated by fluorescence-activated cell sorting from the whole blood of severely injured patients within 12 hours postinjury and the (**A**,**C**) percentage and (**B**,**D**) mean intensity of ROS^{+} and $phagocytosis^{+}$ cells were evaluated. Data are presented as mean ± standard error of the mean.

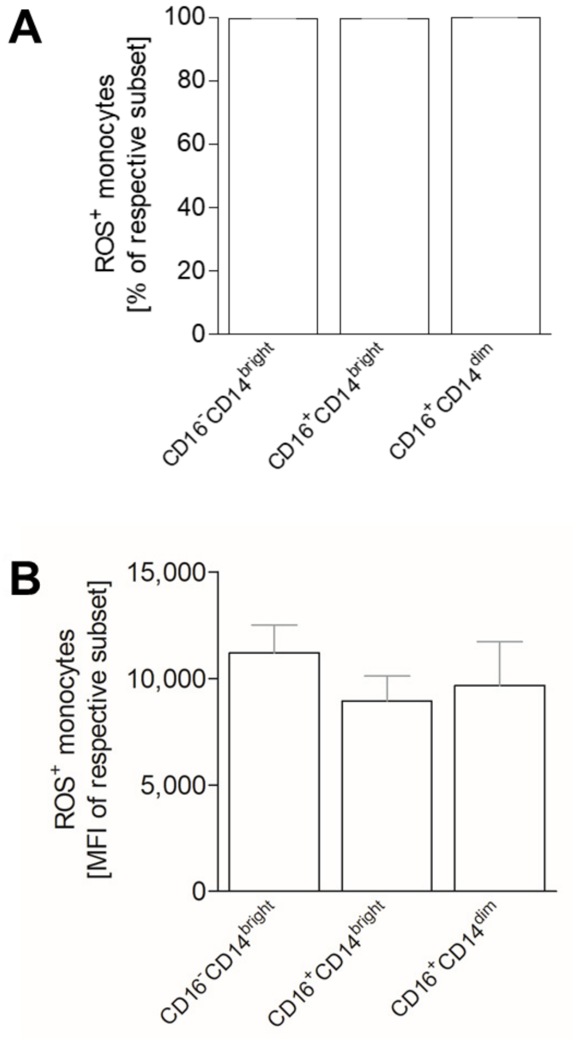

Figure S2. Generation of reactive oxygen species in isolated monocyte subsets obtained from severely injured patients. The $CD14^{bright}CD16^{-}$ (classical), $CD14^{bright}CD16^{+}$ (intermediate) and $CD14^{dim}CD16^{+}$ (non-classical) monocytes were isolated by fluorescence-activated cell sorting from the whole blood of severely injured patients within 12 hours postinjury and the (**A**) percentage and (**B**) mean intensity of ROS^{+} cells were evaluated. Data are presented as mean ± standard error of the mean.
